# Medical Aid Associations

**Published:** 1894-06-02

**Authors:** 


### The Hospital

An Institutional Journal of

The Medical Sciences and Hospital Administration, with

AN EXTRA NURSING SUPPLEMENT.

Vol. XVI.—No. 401.] [June 2, 1894.

### Medical Aid Associations.

The General Council of Medical Education and Registration has once more briefly considered the subject of Medical Aid Associations, but has failed to come to any conclusion except to leave the matter alone for the present. This seems to us to be a very unfortunate conclusion, because, if we mistake not, delay in this, as in so many other things, may prove to be dangerous in certain unexpected directions. The medical aid associations of the immediate present we know, and the extent of the injury they do to the profession and the public we can approximately measure. But, as Shakespeare tells us, the "sight of means to do ill deeds oft makes ill deeds done"; and the example of the medical aid associations which now exist may easily be followed in regions and by persons which neither the Medical Council nor the medical profession as a whole has yet dreamt of.

Those medical men who practice in the dignified seclusion of a West End square, or in the remote wilds of some outlying wold or fen, do not always see exactly what is going on in that heart and centre of modern financial and commercial activity known as the "City of London." But medical men who practice, like the present writer, in the City and among its strenuous money-makers, feel the convulsive heavings of the time spirit in a degree which would seem impossible to their brethren who live on the outer fringes of our modern busy life. It is a well-known fact that remedies of a "quack" order have been put upon the stock market under the auspices of a class of financiers who twenty or thirty years ago would have been ashamed to touch anything of the kind. We could name several such ventures, among the rest, "Sequah's" cures, Warner's Safe Cure, Harness' Belts, and the like. Now those gentlemen who are so ready to finance "quack" remedies would much rather finance a company of half-a-dozen fully-qualified specialists, and share their profits with them. If it should be decided by the General Medical Council that an association of workmen may legitimately employ fully-qualified medical men, advertise their services, and pocket substantial profits, what is to prevent an enterprising syndicate in the City of London or elsewhere taking large, showy premises in a conspicuous position, putting up vast brass plates, advertising in such specious ways as they well know how to advertise, and employing a dozen or a score of medical men, representing every conceivable specialty, and making a splendid profit out of the transaction? If it be said that the public would never be imposed upon by any such scheme, our answer is that the time seems to us to be entirely ripe for just such schemes, that the public would swallow such baits whole, that the thing, if done on a scale sufficiently large and magnificent, would be a brilliant financial success; all the more a success because it would seem to be setting the most cherished traditions of the medical profession at open defiance.

One fact is worth a thousand conjectures in the convincing of the sceptical. Not more than two months ago the head of one of the most eminent firms of accountants in the city of London consulted the present writer about just such a scheme as has been outlined. A medical specialty was to be put upon the market; it was to be worked by qualified medical men; its success as a method of treatment was said to be vouched for by physicians and surgeons of high standing; a company was to be formed, capital raised, and the whole thing launched by means of prospectuses, shareholders, directors, and all the paraphernalia which the company promoter has found to be so effectual in the raising of money a thousand times. The promoters were not profesing to sell a drug, a specific remedy, but to advertise far and wide a method of treatment which qualified and registered practitioners were to carry out. Of course the whole thing was nipped in the bud when it was properly explained to the accountant who had had the bringing out of the scheme offered to him, at any rate so far as he was concerned; and the writer has since heard no more of it. But for anything which is known to the contrary, the scheme may at this moment be maturing in other hands, and any day may see a prospectus on the breakfast tables of the investing public, offering the administration of medical and surgical specialisms by fully qualified medical men in the company's specially designed premises, and with the usual assurances of complete and unfailing success.

A scheme such as is here disclosed may be considered so outrageous as to be practically impossible. No medical man, it may be thought, would be found willing to work it; the Medical Council would put its foot down and stamp it out. But let us ask the medical profession, a profession so innocent of practical business knowledge, and so ignorant of the ways of the financial world, one question: What is the difference in principle between a scheme such as has been here disclosed and some of the medical aid associations which are now in full operation? There is no difference. A medical aid association is for practical purposes a company of laymen; it employs medical men as its servants for wages; it charges for their services, and makes a profit by the transaction. What is the difference, we repeat, between such an association and an association of shareholders and directors who propose to do the very same thing? If there be any difference it is in favour of the shareholders and directors, who would probably employ much more competent medical men than the existing medical aid associations, and would no doubt give them much more liberal pay. In fact, so far as the principles involved are concerned, if the General Medical Council were now to take a false step, there is no reason why the whole medical profession should not in a few years be "run" by clever syndicates of money-making financiers.

Medical aid associations, in our sober judgment, are fraught with danger to the very highest interests of medicine and medical science. At present their insignificance renders them of little or no account from a practical and imperial point of view. But the principles involved are fatal. It seems to us astonishing that so thoughtful a man as Dr. Samuel Wilks should have been so blind to the real issues at stake. Indeed, none of the members of the Council, so far as we can see, saw the whole question in its true light and full significance, Medical aid associations, such as we are discussing, are the "little clouds, no bigger than a man's hand," which have arisen in the serene sky of a responsible and honest medicine, and which threaten to overwhelm both its honesty and its responsibility. Once let them receive the sanction of the General Medical Council, and the mischief will be done never to be undone. Once let richer men take hold of the principle, let them put money into such schemes, let shareholders be brought in and vested interests created, and an evil will be established which all the energy of the whole medical profession will be powerless to disestablish.

No irrevocable step has yet been taken. Let us be thankful for this. Mr. Thomas Bryant and Sir Dyce Duckworth, who were among the majority which rejected the report of the Special Committee, have seen the error of their ways. That Committee would have been re-appointed it Dr. MacAlister had not very unwisely taunted the Council with a desire to eat its own words. The Council was not strong on this point. It had made a mistake; it should have had the courage to unmake the mistake. It must have the courage. In practical politics, as in practical business of all kinds, nothing is so foolish or so fatal as to stand by a mistake for the mere sake of appearing to be consistent. In medical aid associations, as at present conducted, the honour and progress of the medical profession as a whole are placed in jeopardy. If the Medical Council has any proper appreciation of its duty at all it will see to it that that jeopardy does not continue to be a practical factor in the situation for a single unnecessary hour.

